# *N*,*N*′-Bis(4-bromo­phen­yl)naphthalene-1,4-dicarboxamide *N*,*N*-dimethyl­acetamide disolvate

**DOI:** 10.1107/S1600536809051447

**Published:** 2009-12-04

**Authors:** Lin-Hai Jing

**Affiliations:** a School of Chemistry and Chemical Engineering, China West Normal University, Nanchong 637002, People’s Republic of China

## Abstract

The title compound, C_24_H_16_Br_2_N_2_O_2_·2C_4_H_9_NO, crystallizes in an *anti* C=O orientation. The two amide groups are twisted away from the naphthalene ring system by 62.67 (8) and 75.93 (7)°. The crystal packing is stabilized by N—H⋯O and C—H⋯O hydrogen bonds. Each of the dimethyl­acetamide solvent mol­ecules is disordered over two positions, with occupancy ratios of 0.556 (7):0.444 (7) and 0.654 (7):0.346 (7).

## Related literature

For the use of 1,4-naphthalene­dicarboxylic acid derivatives in the preparation of polymers, see: Fukuzumi *et al.* (1994 ▶); Tsukada *et al.* (1994 ▶). For the crystal structure of the 4-methyl­phenyl analog, see: Jing (2008 ▶).
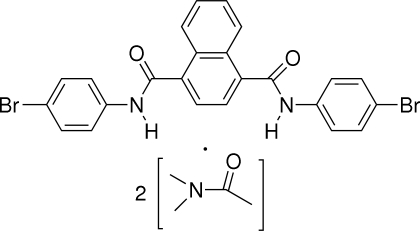


## Experimental

### 

#### Crystal data

C_24_H_16_Br_2_N_2_O_2_·2C_4_H_9_NO
*M* *_r_* = 698.45
Triclinic, 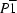

*a* = 10.4589 (9) Å
*b* = 12.4485 (5) Å
*c* = 12.8439 (11) Å
α = 90.775 (2)°
β = 111.370 (4)°
γ = 92.944 (2)°
*V* = 1554.3 (2) Å^3^
*Z* = 2
Mo *K*α radiation
μ = 2.65 mm^−1^
*T* = 93 K
0.47 × 0.33 × 0.17 mm

#### Data collection

Rigaku AFC10/Saturn724+ diffractometer
Absorption correction: multi-scan (*ABSCOR*; Higashi, 1995 ▶) *T*_min_ = 0.364, *T*_max_ = 0.637
12655 measured reflections
6836 independent reflections
5420 reflections with *I* > 2σ(*I*)
*R*_int_ = 0.024

#### Refinement

*R*[*F* ^2^ > 2σ(*F* ^2^)] = 0.029
*wR*(*F* ^2^) = 0.065
*S* = 0.95
6836 reflections
509 parameters
64 restraints
H atoms treated by a mixture of independent and constrained refinement
Δρ_max_ = 0.35 e Å^−3^
Δρ_min_ = −0.73 e Å^−3^

### 

Data collection: *RAPID-AUTO* (Rigaku, 2004 ▶); cell refinement: *RAPID-AUTO*; data reduction: *RAPID-AUTO*; program(s) used to solve structure: *SHELXS97* (Sheldrick, 2008 ▶); program(s) used to refine structure: *SHELXL97* (Sheldrick, 2008 ▶); molecular graphics: *SHELXTL* (Sheldrick, 2008 ▶); software used to prepare material for publication: *SHELXL97*.

## Supplementary Material

Crystal structure: contains datablocks global, I. DOI: 10.1107/S1600536809051447/ci2967sup1.cif

Structure factors: contains datablocks I. DOI: 10.1107/S1600536809051447/ci2967Isup2.hkl

Additional supplementary materials:  crystallographic information; 3D view; checkCIF report

## Figures and Tables

**Table 1:** Hydrogen-bond geometry (Å, °)

*D*—H⋯*A*	*D*—H	H⋯*A*	*D*⋯*A*	*D*—H⋯*A*
N1—H1*N*⋯O4	0.84 (2)	2.03 (3)	2.865 (13)	170 (2)
N2—H2*N*⋯O3′^i^	0.81 (2)	2.00 (3)	2.797 (13)	171 (2)
C2—H2⋯O1^i^	0.95	2.50	3.423 (2)	163
C6—H6⋯O2^ii^	0.95	2.49	3.426 (2)	169
C14—H14⋯O4^iii^	0.95	2.55	3.48 (2)	167

Symmetry codes: (i) 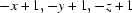; (ii) 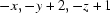; (iii) 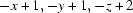.

**Fig. 1.**
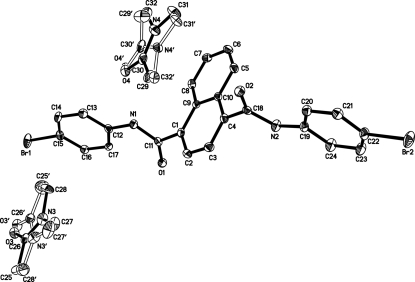
The asymmetric unit of the title compound. Displacement ellipsoids are drawn at the 50% probability level. All disorder components are shown. H atoms have been omitted for clarity.

## supplementary crystallographic information

### Comment

1,4-Naphthalenedicarboxylic acid derivatives are a class of intermediates important for applications as monomers in the preparation of polymers (Fukuzumi *et al.*, 1994; Tsukada *et al.*, 1994). Previously, the author has reported the crystal structure of *N*,*N*'-bis(4-methylphenyl)-1,4-naphthalenedicarboxamide *N*,*N*-dimethylacetamide disolvate (Jing, 2008). The crystal structure of the title compound is now reported.

The naphthalene ring system is planar, with a maximum deviation of 0.030 (3) Å for atom C3. The two C═O groups exhibit an anti orientation. As a result of steric effects, the substituent groups at atoms C1 and C4 are twisted away from the plane of the naphthalene ring system (Fig. 1). The O1/N1/C1/C11 and O2/N2/C4/C18 planes form dihedral angles of 62.67 (8) and 75.93 (7)°, respectively, with the C1–C4/C9/C10 plane. The O1/N1/C1/C11 and C12–C17 planes are inclined at an angle of 16.21 (4)° while the O2/N2/C4/C18 and C19–C24 planes make a dihedral angle of 7.42 (3)°. The crystal packing is stabilized by N—H···O and C—H···O hydrogen bonds (Table 1).

### Experimental

Naphthalene-1,4-dicarboxylic acid (2 mmol) and an excess of thionyl chloride (6 mmol) in dioxane (20 ml) were boiled under reflux for 6 h. The solution was distilled by reduced pressure and a yellow solid was obtained. *p*-Bromoaniline (4 mmol) in tetrahydrofuran (20 ml) was added to the yellow solid and boiled under reflux for 1 d. The solution was then cooled to ambient temperature and filtered to remove the tetrahydrofuran. The precipitate was dissolved in dimethylacetamide and allowed to stand for one month at ambient temperature, after which time colourless single crystals of the title compound suitable for X-ray diffraction were obtained.

### Refinement

Both dimethylacetamide molecules are disordered over two positions. The site-occupation factors for the disordered atoms were refined to 0.444 (7) and 0.556 (7), respectively, for the minor and major components of one of the dimethylacetamide molecules (with O3), and to 0.654 (7) and 0.346 (7), respectively, for the major and minor components of the other molecule. The corresponding distances in the major and minor disorder components were restrained to be the same. The U^ij^ components of atoms O3, O3', C26, C27', C28', C31' and C32'were restrained to an approximate isotropic behaviour. N-bound H atoms were located in a difference Fourier map and refined isotropically (N–H = 0.84 (2) and 0.81 (2) Å). The C-bound H atoms were placed in calculated positions, with C–H = 0.95 or 0.98 Å, and refined using a riding model, with *U*_iso_(H) = 1.2*U*_eq_(C).

### Crystal data

**Table:**

C_24_H_16_Br_2_N_2_O_2_·2C_4_H_9_NO	*Z* = 2
*M**_r_* = 698.45	*F*(000) = 712
Triclinic, *P*1	*D*_x_ = 1.492 Mg m^−^^3^
Hall symbol: -P 1	Mo *K*α radiation, λ = 0.71073 Å
*a* = 10.4589 (9) Å	Cell parameters from 4940 reflections
*b* = 12.4485 (5) Å	θ = 3.2–27.5°
*c* = 12.8439 (11) Å	µ = 2.65 mm^−^^1^
α = 90.775 (2)°	*T* = 93 K
β = 111.370 (4)°	Prism, colourless
γ = 92.944 (2)°	0.47 × 0.33 × 0.17 mm
*V* = 1554.3 (2) Å^3^	

### Data collection

**Table:**

Rigaku AFC10/Saturn724+ diffractometer	6836 independent reflections
Radiation source: Rotating Anode	5420 reflections with *I* > 2σ(*I*)
graphite	*R*_int_ = 0.024
Detector resolution: 28.5714 pixels mm^-1^	θ_max_ = 27.5°, θ_min_ = 3.2°
ω scans	*h* = −11→13
Absorption correction: multi-scan (*ABSCOR*; Higashi, 1995)	*k* = −15→16
*T*_min_ = 0.364, *T*_max_ = 0.637	*l* = −16→14
12655 measured reflections	

### Refinement

**Table:**

Refinement on *F*^2^	Primary atom site location: structure-invariant direct methods
Least-squares matrix: full	Secondary atom site location: difference Fourier map
*R*[*F*^2^ > 2σ(*F*^2^)] = 0.029	Hydrogen site location: inferred from neighbouring sites
*wR*(*F*^2^) = 0.065	H atoms treated by a mixture of independent and constrained refinement
*S* = 0.95	*w* = 1/[σ^2^(*F*_o_^2^) + (0.0312*P*)^2^] where *P* = (*F*_o_^2^ + 2*F*_c_^2^)/3
6836 reflections	(Δ/σ)_max_ = 0.001
509 parameters	Δρ_max_ = 0.35 e Å^−^^3^
64 restraints	Δρ_min_ = −0.72 e Å^−^^3^

### Special details

**Table:**

Geometry. All e.s.d.'s (except the e.s.d. in the dihedral angle between two l.s. planes) are estimated using the full covariance matrix. The cell e.s.d.'s are taken into account individually in the estimation of e.s.d.'s in distances, angles and torsion angles; correlations between e.s.d.'s in cell parameters are only used when they are defined by crystal symmetry. An approximate (isotropic) treatment of cell e.s.d.'s is used for estimating e.s.d.'s involving l.s. planes.
Refinement. Refinement of *F*^2^ against ALL reflections. The weighted *R*-factor *wR* and goodness of fit *S* are based on *F*^2^, conventional *R*-factors *R* are based on *F*, with *F* set to zero for negative *F*^2^. The threshold expression of *F*^2^ > σ(*F*^2^) is used only for calculating *R*-factors(gt) *etc*. and is not relevant to the choice of reflections for refinement. *R*-factors based on *F*^2^ are statistically about twice as large as those based on *F*, and *R*- factors based on ALL data will be even larger.

### Fractional atomic coordinates and isotropic or equivalent isotropic displacement parameters (Å^2^)

**Table:**

	*x*	*y*	*z*	*U*_iso_*/*U*_eq_	Occ. (<1)
Br1	0.53236 (2)	0.112534 (16)	0.932514 (17)	0.02903 (7)	
Br2	0.22415 (2)	1.394801 (16)	0.057404 (18)	0.03060 (7)	
O1	0.32331 (13)	0.45436 (10)	0.48313 (11)	0.0177 (3)	
O2	0.31551 (13)	1.04379 (10)	0.50572 (11)	0.0191 (3)	
N1	0.34837 (17)	0.50996 (13)	0.66106 (14)	0.0168 (3)	
H1N	0.348 (2)	0.5650 (19)	0.700 (2)	0.039 (7)*	
N2	0.23601 (17)	0.99105 (13)	0.32026 (14)	0.0189 (4)	
H2N	0.203 (2)	0.9395 (17)	0.2781 (19)	0.024 (6)*	
C1	0.30036 (18)	0.63948 (14)	0.51685 (15)	0.0143 (4)	
C2	0.39398 (19)	0.68975 (15)	0.47878 (16)	0.0171 (4)	
H2	0.4670	0.6508	0.4729	0.021*	
C3	0.38368 (19)	0.79752 (15)	0.44845 (16)	0.0172 (4)	
H3	0.4495	0.8307	0.4222	0.021*	
C4	0.27922 (18)	0.85564 (14)	0.45630 (15)	0.0145 (4)	
C5	0.06363 (19)	0.86155 (15)	0.49518 (16)	0.0170 (4)	
H5	0.0559	0.9348	0.4750	0.020*	
C6	−0.03434 (19)	0.81135 (15)	0.52719 (16)	0.0183 (4)	
H6	−0.1095	0.8499	0.5291	0.022*	
C7	−0.02522 (19)	0.70304 (15)	0.55738 (16)	0.0178 (4)	
H7	−0.0940	0.6690	0.5798	0.021*	
C8	0.08240 (18)	0.64638 (15)	0.55463 (16)	0.0159 (4)	
H8	0.0872	0.5731	0.5746	0.019*	
C9	0.18715 (18)	0.69610 (14)	0.52222 (15)	0.0130 (4)	
C10	0.17723 (18)	0.80569 (14)	0.49159 (15)	0.0136 (4)	
C11	0.32325 (18)	0.52500 (14)	0.55106 (16)	0.0144 (4)	
C12	0.38661 (19)	0.41416 (14)	0.71995 (16)	0.0153 (4)	
C13	0.44442 (19)	0.42461 (15)	0.83616 (16)	0.0184 (4)	
H13	0.4552	0.4937	0.8717	0.022*	
C14	0.48654 (19)	0.33506 (15)	0.90073 (16)	0.0187 (4)	
H14	0.5262	0.3420	0.9800	0.022*	
C15	0.46910 (19)	0.23536 (15)	0.84642 (16)	0.0175 (4)	
C16	0.40962 (18)	0.22272 (14)	0.73121 (16)	0.0158 (4)	
H16	0.3972	0.1532	0.6962	0.019*	
C17	0.36833 (18)	0.31242 (14)	0.66737 (16)	0.0145 (4)	
H17	0.3278	0.3048	0.5882	0.017*	
C18	0.27844 (18)	0.97352 (14)	0.43114 (16)	0.0147 (4)	
C19	0.23255 (19)	1.08886 (15)	0.26482 (16)	0.0162 (4)	
C20	0.27827 (18)	1.18916 (15)	0.31961 (16)	0.0169 (4)	
H20	0.3117	1.1949	0.3990	0.020*	
C21	0.27480 (19)	1.28043 (15)	0.25775 (16)	0.0180 (4)	
H21	0.3053	1.3490	0.2944	0.022*	
C22	0.2264 (2)	1.27038 (15)	0.14216 (17)	0.0195 (4)	
C23	0.1782 (2)	1.17201 (15)	0.08598 (17)	0.0236 (5)	
H23	0.1433	1.1669	0.0066	0.028*	
C24	0.1820 (2)	1.08126 (15)	0.14807 (17)	0.0222 (4)	
H24	0.1500	1.0132	0.1108	0.027*	
O3	0.9394 (16)	0.1698 (9)	0.8232 (17)	0.021 (2)	0.444 (7)
N3	0.8990 (5)	0.3438 (4)	0.7831 (4)	0.0226 (14)	0.444 (7)
C25	1.0986 (17)	0.2599 (10)	0.7668 (15)	0.025 (2)	0.444 (7)
H25A	1.1649	0.3154	0.8142	0.030*	0.444 (7)
H25B	1.0762	0.2764	0.6879	0.030*	0.444 (7)
H25C	1.1387	0.1896	0.7810	0.030*	0.444 (7)
C26	0.9712 (6)	0.2573 (4)	0.7930 (4)	0.0205 (15)	0.444 (7)
C27	0.9413 (14)	0.4520 (8)	0.7573 (15)	0.024 (2)	0.444 (7)
H27A	0.9882	0.4942	0.8271	0.029*	0.444 (7)
H27B	0.8599	0.4881	0.7108	0.029*	0.444 (7)
H27C	1.0041	0.4454	0.7168	0.029*	0.444 (7)
C28	0.7799 (19)	0.3420 (14)	0.8162 (18)	0.024 (3)	0.444 (7)
H28A	0.7246	0.2743	0.7893	0.029*	0.444 (7)
H28B	0.7242	0.4026	0.7837	0.029*	0.444 (7)
H28C	0.8107	0.3478	0.8980	0.029*	0.444 (7)
O3'	0.9084 (13)	0.1781 (7)	0.8283 (14)	0.0227 (19)	0.556 (7)
N3'	0.9840 (5)	0.3352 (3)	0.7788 (3)	0.0245 (12)	0.556 (7)
C25'	0.7828 (18)	0.3251 (12)	0.8320 (16)	0.040 (4)	0.556 (7)
H25D	0.7252	0.2693	0.8500	0.048*	0.556 (7)
H25E	0.7272	0.3612	0.7646	0.048*	0.556 (7)
H25F	0.8198	0.3778	0.8946	0.048*	0.556 (7)
C26'	0.8982 (4)	0.2748 (4)	0.8120 (3)	0.0197 (12)	0.556 (7)
C27'	0.9802 (11)	0.4515 (7)	0.7669 (13)	0.036 (3)	0.556 (7)
H27D	0.9253	0.4673	0.6894	0.043*	0.556 (7)
H27E	1.0740	0.4832	0.7864	0.043*	0.556 (7)
H27F	0.9387	0.4821	0.8169	0.043*	0.556 (7)
C28'	1.0974 (13)	0.2903 (8)	0.7562 (12)	0.034 (3)	0.556 (7)
H28D	1.1842	0.3113	0.8174	0.041*	0.556 (7)
H28E	1.1003	0.3177	0.6858	0.041*	0.556 (7)
H28F	1.0841	0.2116	0.7501	0.041*	0.556 (7)
O4	0.3394 (12)	0.6785 (6)	0.8119 (18)	0.0205 (19)	0.654 (7)
N4	0.2121 (3)	0.8208 (3)	0.8085 (2)	0.0229 (10)	0.654 (7)
C29	0.4147 (11)	0.8427 (10)	0.7597 (11)	0.028 (3)	0.654 (7)
H29A	0.4752	0.8870	0.8243	0.033*	0.654 (7)
H29B	0.3635	0.8896	0.7000	0.033*	0.654 (7)
H29C	0.4701	0.7967	0.7327	0.033*	0.654 (7)
C30	0.3170 (4)	0.7747 (3)	0.7932 (3)	0.0205 (10)	0.654 (7)
C31	0.1860 (9)	0.9350 (5)	0.7976 (8)	0.0283 (17)	0.654 (7)
H31A	0.2042	0.9679	0.8717	0.034*	0.654 (7)
H31B	0.0898	0.9430	0.7499	0.034*	0.654 (7)
H31C	0.2465	0.9706	0.7639	0.034*	0.654 (7)
C32	0.1180 (15)	0.7532 (12)	0.8495 (11)	0.030 (2)	0.654 (7)
H32A	0.1453	0.6787	0.8559	0.036*	0.654 (7)
H32B	0.0231	0.7554	0.7965	0.036*	0.654 (7)
H32C	0.1243	0.7814	0.9229	0.036*	0.654 (7)
O4'	0.312 (2)	0.6646 (12)	0.810 (3)	0.019 (3)	0.346 (7)
N4'	0.2828 (6)	0.8424 (5)	0.7762 (4)	0.0184 (18)	0.346 (7)
C29'	0.142 (3)	0.748 (2)	0.858 (2)	0.034 (5)	0.346 (7)
H29D	0.1246	0.6751	0.8791	0.041*	0.346 (7)
H29E	0.0585	0.7721	0.8007	0.041*	0.346 (7)
H29F	0.1708	0.7969	0.9235	0.041*	0.346 (7)
C30'	0.2524 (8)	0.7475 (5)	0.8120 (5)	0.022 (2)	0.346 (7)
C31'	0.2132 (17)	0.9405 (10)	0.7792 (16)	0.023 (3)	0.346 (7)
H31D	0.1200	0.9215	0.7765	0.028*	0.346 (7)
H31E	0.2084	0.9836	0.7147	0.028*	0.346 (7)
H31F	0.2647	0.9823	0.8484	0.028*	0.346 (7)
C32'	0.4106 (17)	0.854 (2)	0.746 (2)	0.019 (4)	0.346 (7)
H32D	0.4930	0.8586	0.8142	0.023*	0.346 (7)
H32E	0.4078	0.9197	0.7038	0.023*	0.346 (7)
H32F	0.4132	0.7915	0.6995	0.023*	0.346 (7)

### Atomic displacement parameters (Å^2^)

**Table:**

	*U*^11^	*U*^22^	*U*^33^	*U*^12^	*U*^13^	*U*^23^
Br1	0.04347 (14)	0.01313 (10)	0.01985 (11)	0.00236 (9)	−0.00130 (10)	0.00551 (8)
Br2	0.05477 (16)	0.01552 (11)	0.02162 (12)	0.00101 (10)	0.01403 (11)	0.00613 (8)
O1	0.0235 (7)	0.0134 (7)	0.0165 (7)	0.0034 (5)	0.0073 (6)	0.0003 (5)
O2	0.0257 (7)	0.0144 (7)	0.0169 (7)	0.0017 (6)	0.0075 (6)	−0.0002 (5)
N1	0.0254 (9)	0.0102 (8)	0.0142 (8)	0.0058 (7)	0.0059 (7)	0.0008 (6)
N2	0.0279 (10)	0.0105 (8)	0.0154 (9)	−0.0011 (7)	0.0050 (8)	−0.0003 (7)
C1	0.0167 (9)	0.0128 (9)	0.0113 (9)	0.0024 (7)	0.0023 (8)	0.0013 (7)
C2	0.0178 (10)	0.0165 (10)	0.0185 (10)	0.0055 (8)	0.0078 (8)	0.0022 (8)
C3	0.0172 (10)	0.0175 (10)	0.0186 (10)	0.0008 (8)	0.0089 (8)	0.0018 (8)
C4	0.0158 (9)	0.0129 (9)	0.0139 (9)	0.0018 (7)	0.0041 (8)	0.0015 (7)
C5	0.0186 (10)	0.0135 (9)	0.0181 (10)	0.0041 (8)	0.0051 (8)	0.0022 (7)
C6	0.0148 (10)	0.0201 (10)	0.0206 (10)	0.0043 (8)	0.0068 (8)	0.0000 (8)
C7	0.0174 (10)	0.0191 (10)	0.0193 (10)	0.0000 (8)	0.0096 (8)	0.0007 (8)
C8	0.0157 (10)	0.0144 (9)	0.0159 (10)	0.0011 (7)	0.0037 (8)	0.0028 (7)
C9	0.0160 (9)	0.0128 (9)	0.0096 (9)	0.0025 (7)	0.0037 (8)	−0.0009 (7)
C10	0.0143 (9)	0.0135 (9)	0.0116 (9)	0.0017 (7)	0.0031 (8)	0.0001 (7)
C11	0.0116 (9)	0.0142 (9)	0.0169 (10)	0.0024 (7)	0.0043 (8)	0.0023 (7)
C12	0.0159 (10)	0.0132 (9)	0.0174 (10)	0.0041 (7)	0.0062 (8)	0.0036 (7)
C13	0.0227 (10)	0.0130 (9)	0.0181 (10)	0.0024 (8)	0.0057 (8)	−0.0013 (7)
C14	0.0223 (10)	0.0179 (10)	0.0121 (9)	0.0009 (8)	0.0018 (8)	0.0012 (7)
C15	0.0200 (10)	0.0121 (9)	0.0192 (10)	0.0039 (8)	0.0051 (8)	0.0054 (8)
C16	0.0173 (10)	0.0117 (9)	0.0189 (10)	0.0002 (7)	0.0074 (8)	0.0010 (7)
C17	0.0161 (10)	0.0151 (9)	0.0130 (9)	0.0021 (7)	0.0058 (8)	0.0009 (7)
C18	0.0127 (9)	0.0145 (9)	0.0193 (10)	0.0031 (7)	0.0083 (8)	0.0038 (7)
C19	0.0191 (10)	0.0135 (9)	0.0164 (10)	0.0025 (8)	0.0065 (8)	0.0040 (7)
C20	0.0176 (10)	0.0169 (10)	0.0150 (10)	0.0015 (8)	0.0043 (8)	0.0013 (7)
C21	0.0213 (10)	0.0128 (9)	0.0197 (10)	−0.0001 (8)	0.0073 (9)	−0.0003 (7)
C22	0.0257 (11)	0.0135 (9)	0.0204 (10)	0.0026 (8)	0.0094 (9)	0.0061 (8)
C23	0.0352 (12)	0.0186 (10)	0.0154 (10)	0.0018 (9)	0.0074 (9)	0.0020 (8)
C24	0.0328 (12)	0.0121 (9)	0.0186 (10)	−0.0015 (8)	0.0062 (9)	−0.0011 (8)
O3	0.023 (5)	0.017 (3)	0.022 (3)	0.002 (2)	0.004 (3)	−0.001 (2)
N3	0.024 (3)	0.017 (3)	0.023 (2)	−0.006 (2)	0.006 (2)	0.0007 (18)
C25	0.027 (4)	0.026 (5)	0.025 (4)	−0.003 (4)	0.014 (3)	−0.007 (4)
C26	0.019 (3)	0.029 (4)	0.009 (2)	−0.001 (2)	0.000 (2)	−0.0051 (19)
C27	0.026 (5)	0.019 (4)	0.022 (4)	−0.004 (3)	0.004 (5)	0.000 (3)
C28	0.017 (5)	0.022 (4)	0.036 (6)	0.001 (3)	0.011 (4)	−0.011 (5)
O3'	0.028 (5)	0.014 (2)	0.022 (2)	0.001 (2)	0.004 (3)	−0.0015 (18)
N3'	0.028 (3)	0.024 (2)	0.0216 (18)	−0.0096 (17)	0.0109 (17)	−0.0037 (14)
C25'	0.036 (5)	0.040 (6)	0.047 (7)	−0.002 (4)	0.018 (4)	0.001 (4)
C26'	0.019 (3)	0.022 (3)	0.0159 (19)	−0.0001 (18)	0.0043 (17)	−0.0034 (17)
C27'	0.044 (6)	0.027 (3)	0.029 (4)	−0.013 (3)	0.007 (5)	0.009 (3)
C28'	0.022 (3)	0.050 (6)	0.028 (4)	−0.005 (4)	0.007 (3)	−0.008 (4)
O4	0.023 (4)	0.015 (2)	0.018 (2)	0.002 (2)	0.001 (4)	−0.003 (2)
N4	0.0200 (17)	0.0206 (19)	0.0267 (16)	0.0044 (13)	0.0065 (13)	−0.0019 (12)
C29	0.036 (4)	0.029 (4)	0.017 (3)	0.000 (3)	0.009 (3)	−0.004 (3)
C30	0.022 (2)	0.022 (3)	0.0114 (16)	0.0008 (18)	−0.0018 (15)	−0.0035 (14)
C31	0.021 (3)	0.020 (2)	0.040 (4)	0.006 (2)	0.007 (3)	−0.005 (2)
C32	0.021 (4)	0.047 (4)	0.024 (3)	0.000 (3)	0.011 (3)	0.006 (3)
O4'	0.018 (7)	0.014 (4)	0.020 (4)	0.003 (5)	0.001 (6)	−0.008 (5)
N4'	0.020 (3)	0.015 (3)	0.018 (3)	0.005 (2)	0.004 (2)	−0.003 (2)
C29'	0.018 (8)	0.040 (9)	0.041 (9)	−0.013 (6)	0.010 (6)	−0.019 (7)
C30'	0.021 (4)	0.025 (5)	0.012 (3)	−0.004 (4)	−0.002 (3)	−0.001 (3)
C31'	0.018 (6)	0.020 (4)	0.032 (5)	0.008 (4)	0.008 (4)	0.002 (3)
C32'	0.008 (5)	0.027 (6)	0.022 (7)	−0.005 (4)	0.005 (4)	0.006 (4)

### Geometric parameters (Å, °)

**Table:**

Br1—C15	1.9013 (18)	C25—C26	1.487 (16)
Br2—C22	1.9014 (18)	C25—H25A	0.98
O1—C11	1.231 (2)	C25—H25B	0.98
O2—C18	1.227 (2)	C25—H25C	0.98
N1—C11	1.357 (2)	C27—H27A	0.98
N1—C12	1.417 (2)	C27—H27B	0.98
N1—H1N	0.84 (2)	C27—H27C	0.98
N2—C18	1.353 (2)	C28—H28A	0.98
N2—C19	1.414 (2)	C28—H28B	0.98
N2—H2N	0.81 (2)	C28—H28C	0.98
C1—C2	1.372 (2)	O3'—C26'	1.229 (8)
C1—C9	1.431 (2)	N3'—C26'	1.330 (6)
C1—C11	1.502 (2)	N3'—C28'	1.457 (13)
C2—C3	1.401 (2)	N3'—C27'	1.458 (8)
C2—H2	0.95	C25'—C26'	1.488 (14)
C3—C4	1.374 (3)	C25'—H25D	0.98
C3—H3	0.95	C25'—H25E	0.98
C4—C10	1.422 (3)	C25'—H25F	0.98
C4—C18	1.507 (2)	C27'—H27D	0.98
C5—C6	1.363 (3)	C27'—H27E	0.98
C5—C10	1.422 (2)	C27'—H27F	0.98
C5—H5	0.95	C28'—H28D	0.98
C6—C7	1.406 (3)	C28'—H28E	0.98
C6—H6	0.95	C28'—H28F	0.98
C7—C8	1.369 (3)	O4—C30	1.242 (8)
C7—H7	0.95	N4—C30	1.337 (5)
C8—C9	1.423 (3)	N4—C31	1.460 (7)
C8—H8	0.95	N4—C32	1.503 (11)
C9—C10	1.423 (2)	C29—C30	1.479 (11)
C12—C13	1.393 (3)	C29—H29A	0.98
C12—C17	1.398 (3)	C29—H29B	0.98
C13—C14	1.391 (2)	C29—H29C	0.98
C13—H13	0.95	C30—O4	1.242 (8)
C14—C15	1.386 (3)	C31—H31A	0.98
C14—H14	0.95	C31—H31B	0.98
C15—C16	1.384 (3)	C31—H31C	0.98
C16—C17	1.386 (2)	C32—H32A	0.98
C16—H16	0.95	C32—H32B	0.98
C17—H17	0.95	C32—H32C	0.98
C19—C24	1.397 (3)	O4'—C30'	1.236 (13)
C19—C20	1.397 (3)	N4'—C30'	1.338 (9)
C20—C21	1.390 (2)	N4'—C31'	1.461 (11)
C20—H20	0.95	N4'—C32'	1.525 (16)
C21—C22	1.384 (3)	C29'—C30'	1.468 (18)
C21—H21	0.95	C29'—H29D	0.98
C22—C23	1.386 (3)	C29'—H29E	0.98
C23—C24	1.385 (3)	C29'—H29F	0.98
C23—H23	0.95	C31'—H31D	0.98
C24—H24	0.95	C31'—H31E	0.98
O3—C26	1.231 (10)	C31'—H31F	0.98
N3—C26	1.328 (8)	C32'—H32D	0.98
N3—C28	1.455 (16)	C32'—H32E	0.98
N3—C27	1.476 (10)	C32'—H32F	0.98
			
C11—N1—C12	127.23 (16)	C21—C22—Br2	119.34 (15)
C11—N1—H1N	116.2 (16)	C23—C22—Br2	118.80 (15)
C12—N1—H1N	116.3 (16)	C24—C23—C22	118.63 (19)
C18—N2—C19	129.45 (17)	C24—C23—H23	120.7
C18—N2—H2N	117.0 (15)	C22—C23—H23	120.7
C19—N2—H2N	113.5 (15)	C23—C24—C19	120.67 (18)
C2—C1—C9	119.96 (16)	C23—C24—H24	119.7
C2—C1—C11	116.99 (15)	C19—C24—H24	119.7
C9—C1—C11	123.05 (15)	C26—N3—C28	120.6 (9)
C1—C2—C3	121.09 (16)	C26—N3—C27	124.9 (8)
C1—C2—H2	119.5	C28—N3—C27	113.7 (10)
C3—C2—H2	119.5	O3—C26—N3	123.8 (9)
C4—C3—C2	120.59 (17)	O3—C26—C25	114.8 (9)
C4—C3—H3	119.7	N3—C26—C25	121.3 (7)
C2—C3—H3	119.7	C26'—N3'—C28'	122.0 (6)
C3—C4—C10	120.19 (16)	C26'—N3'—C27'	124.8 (7)
C3—C4—C18	118.68 (16)	C28'—N3'—C27'	113.1 (7)
C10—C4—C18	121.09 (15)	C26'—C25'—H25D	109.6
C6—C5—C10	120.79 (16)	C26'—C25'—H25E	109.5
C6—C5—H5	119.6	H25D—C25'—H25E	109.5
C10—C5—H5	119.6	C26'—C25'—H25F	109.3
C5—C6—C7	120.67 (16)	H25D—C25'—H25F	109.5
C5—C6—H6	119.7	H25E—C25'—H25F	109.5
C7—C6—H6	119.7	O3'—C26'—N3'	123.7 (7)
C8—C7—C6	120.29 (17)	O3'—C26'—C25'	116.7 (9)
C8—C7—H7	119.9	N3'—C26'—C25'	119.5 (8)
C6—C7—H7	119.9	N3'—C27'—H27D	109.5
C7—C8—C9	120.74 (16)	N3'—C27'—H27E	109.5
C7—C8—H8	119.6	H27D—C27'—H27E	109.5
C9—C8—H8	119.6	N3'—C27'—H27F	109.5
C8—C9—C10	118.71 (15)	H27D—C27'—H27F	109.5
C8—C9—C1	122.53 (16)	H27E—C27'—H27F	109.5
C10—C9—C1	118.74 (15)	N3'—C28'—H28D	109.5
C4—C10—C5	121.84 (16)	N3'—C28'—H28E	109.5
C4—C10—C9	119.35 (15)	H28D—C28'—H28E	109.5
C5—C10—C9	118.80 (15)	N3'—C28'—H28F	109.4
O1—C11—N1	125.01 (17)	H28D—C28'—H28F	109.5
O1—C11—C1	120.76 (16)	H28E—C28'—H28F	109.5
N1—C11—C1	114.18 (16)	C30—N4—C31	125.5 (5)
C13—C12—C17	119.83 (16)	C30—N4—C32	118.6 (7)
C13—C12—N1	116.77 (17)	C31—N4—C32	115.7 (7)
C17—C12—N1	123.41 (17)	O4—C30—N4	122.4 (8)
C14—C13—C12	120.73 (18)	O4—C30—N4	122.4 (8)
C14—C13—H13	119.6	O4—C30—C29	118.9 (9)
C12—C13—H13	119.6	O4—C30—C29	118.9 (9)
C15—C14—C13	118.27 (18)	N4—C30—C29	118.6 (6)
C15—C14—H14	120.9	C30'—N4'—C31'	124.2 (8)
C13—C14—H14	120.9	C30'—N4'—C32'	118.4 (11)
C16—C15—C14	122.03 (17)	C31'—N4'—C32'	116.8 (11)
C16—C15—Br1	118.91 (14)	C30'—C29'—H29D	109.5
C14—C15—Br1	119.05 (15)	C30'—C29'—H29E	109.4
C15—C16—C17	119.36 (17)	H29D—C29'—H29E	109.5
C15—C16—H16	120.3	C30'—C29'—H29F	109.5
C17—C16—H16	120.3	H29D—C29'—H29F	109.5
C16—C17—C12	119.76 (17)	H29E—C29'—H29F	109.5
C16—C17—H17	120.1	O4'—C30'—N4'	124.1 (16)
C12—C17—H17	120.1	O4'—C30'—C29'	120.9 (18)
O2—C18—N2	125.32 (17)	N4'—C30'—C29'	115.0 (12)
O2—C18—C4	121.85 (17)	N4'—C31'—H31D	109.5
N2—C18—C4	112.82 (16)	N4'—C31'—H31E	109.5
C24—C19—C20	119.70 (17)	H31D—C31'—H31E	109.5
C24—C19—N2	116.18 (17)	N4'—C31'—H31F	109.5
C20—C19—N2	124.11 (17)	H31D—C31'—H31F	109.5
C21—C20—C19	119.86 (18)	H31E—C31'—H31F	109.5
C21—C20—H20	120.1	N4'—C32'—H32D	109.5
C19—C20—H20	120.1	N4'—C32'—H32E	109.5
C22—C21—C20	119.26 (18)	H32D—C32'—H32E	109.5
C22—C21—H21	120.4	N4'—C32'—H32F	109.5
C20—C21—H21	120.4	H32D—C32'—H32F	109.5
C21—C22—C23	121.86 (17)	H32E—C32'—H32F	109.5
			
C9—C1—C2—C3	2.3 (3)	C13—C12—C17—C16	−0.9 (3)
C11—C1—C2—C3	−177.90 (18)	N1—C12—C17—C16	179.49 (16)
C1—C2—C3—C4	0.1 (3)	C19—N2—C18—O2	4.7 (3)
C2—C3—C4—C10	−2.3 (3)	C19—N2—C18—C4	−174.05 (17)
C2—C3—C4—C18	175.41 (18)	C3—C4—C18—O2	−102.4 (2)
C10—C5—C6—C7	0.0 (3)	C10—C4—C18—O2	75.2 (2)
C5—C6—C7—C8	−0.2 (3)	C3—C4—C18—N2	76.3 (2)
C6—C7—C8—C9	0.5 (3)	C10—C4—C18—N2	−106.0 (2)
C7—C8—C9—C10	−0.6 (3)	C18—N2—C19—C24	−179.70 (19)
C7—C8—C9—C1	−179.19 (18)	C18—N2—C19—C20	1.6 (3)
C2—C1—C9—C8	176.20 (17)	C24—C19—C20—C21	−0.8 (3)
C11—C1—C9—C8	−3.6 (3)	N2—C19—C20—C21	177.91 (17)
C2—C1—C9—C10	−2.4 (3)	C19—C20—C21—C22	−0.3 (3)
C11—C1—C9—C10	177.81 (17)	C20—C21—C22—C23	1.5 (3)
C3—C4—C10—C5	−176.67 (18)	C20—C21—C22—Br2	−179.05 (13)
C18—C4—C10—C5	5.7 (3)	C21—C22—C23—C24	−1.5 (3)
C3—C4—C10—C9	2.1 (3)	Br2—C22—C23—C24	179.00 (15)
C18—C4—C10—C9	−175.54 (17)	C22—C23—C24—C19	0.4 (3)
C6—C5—C10—C4	178.69 (18)	C20—C19—C24—C23	0.7 (3)
C6—C5—C10—C9	−0.1 (3)	N2—C19—C24—C23	−178.07 (18)
C8—C9—C10—C4	−178.42 (17)	C28—N3—C26—O3	4.5 (17)
C1—C9—C10—C4	0.2 (3)	C27—N3—C26—O3	174.0 (15)
C8—C9—C10—C5	0.4 (3)	C28—N3—C26—C25	−175.8 (13)
C1—C9—C10—C5	179.02 (17)	C27—N3—C26—C25	−6.4 (13)
C12—N1—C11—O1	3.9 (3)	C28'—N3'—C26'—O3'	1.5 (13)
C12—N1—C11—C1	−173.45 (16)	C27'—N3'—C26'—O3'	−175.2 (12)
C2—C1—C11—O1	−61.0 (2)	C28'—N3'—C26'—C25'	−179.0 (12)
C9—C1—C11—O1	118.8 (2)	C27'—N3'—C26'—C25'	4.3 (12)
C2—C1—C11—N1	116.43 (19)	O4—O4—C30—N4	0(3)
C9—C1—C11—N1	−63.8 (2)	O4—O4—C30—C29	0(4)
C11—N1—C12—C13	162.24 (18)	C31—N4—C30—O4	174.5 (12)
C11—N1—C12—C17	−18.1 (3)	C32—N4—C30—O4	−0.1 (13)
C17—C12—C13—C14	1.1 (3)	C31—N4—C30—O4	174.5 (12)
N1—C12—C13—C14	−179.22 (16)	C32—N4—C30—O4	−0.1 (13)
C12—C13—C14—C15	−0.2 (3)	C31—N4—C30—C29	−1.6 (9)
C13—C14—C15—C16	−1.0 (3)	C32—N4—C30—C29	−176.2 (9)
C13—C14—C15—Br1	177.59 (14)	C31'—N4'—C30'—O4'	−180 (2)
C14—C15—C16—C17	1.3 (3)	C32'—N4'—C30'—O4'	−9(2)
Br1—C15—C16—C17	−177.35 (13)	C31'—N4'—C30'—C29'	−0.9 (17)
C15—C16—C17—C12	−0.3 (3)	C32'—N4'—C30'—C29'	170.0 (16)

### Hydrogen-bond geometry (Å, °)

**Table:**

*D*—H···*A*	*D*—H	H···*A*	*D*···*A*	*D*—H···*A*
N1—H1N···O4	0.84 (2)	2.03 (3)	2.865 (13)	170 (2)
N2—H2N···O3'^i^	0.81 (2)	2.00 (3)	2.797 (13)	171 (2)
C2—H2···O1^i^	0.95	2.50	3.423 (2)	163
C6—H6···O2^ii^	0.95	2.49	3.426 (2)	169
C14—H14···O4^iii^	0.95	2.55	3.48 (2)	167

Symmetry codes: (i) −*x*+1, −*y*+1, −*z*+1; (ii) −*x*, −*y*+2, −*z*+1; (iii) −*x*+1, −*y*+1, −*z*+2.
